# Dissipation Behavior and Dietary Risk of Etofenprox in Kale (*Brassica oleracea*) and Red Mustard Greens (*Brassica juncea*)

**DOI:** 10.3390/foods14244223

**Published:** 2025-12-09

**Authors:** Jae-Hyeong Kim, Hye-Min Kwak, Ga-Eul-Hae An, Joon-Kyung Oh, Hee-Ra Chang

**Affiliations:** 1 Department of Pharmaceutical Engineering, Graduate School of Hoseo University, Asan 31499, Republic of Korea; jaebro99@naver.com (J.-H.K.); nofallsun1004@naver.com (G.-E.-H.A.); 555wnsrud@naver.com (J.-K.O.); 2 Pesticide and Veterinary Drug Residues Divisions, National Institute of Food and Drug Safety Evaluation, Ministry of Food and Drug Safety, Osong 28159, Republic of Korea; rhkr1235@gmail.com

**Keywords:** dietary risk assessment, dissipation kinetics, leafy vegetables, MRLs, pesticide

## Abstract

This study evaluated the dissipation kinetics and dietary risk of etofenprox in kale (*Brassica oleracea*) and red mustard greens (*Brassica juncea*), leafy vegetables frequently reported to exceed residue limits in Korea. Field trials were conducted at three sites, and residues were analyzed using QuEChERS extraction followed by LC–MS/MS in accordance with MFDS and SANTE guidelines. The method validation parameters—specificity, linearity, limit of quantitation, accuracy, and precision—were within the acceptable criteria specified by the guidelines. The half-lives of etofenprox under greenhouse conditions were 2.2 days in kale and 3.1 days in red mustard greens, with dissipation rate constants of 0.3118 and 0.2232, respectively. Dietary risk assessment based on residue levels and consumption data confirmed that the %ADI values at the pre-harvest interval (PHI, 7 days) for were <1% the average consumer group and <4% for the high-intake group. Accordingly, the residue levels were considered safe, indicating that compliance with recommended application practices poses negligible health risk to consumers.

## 1. Introduction

Ensuring food safety through effective management of pesticide residues in agricultural products remains a critical global challenge. In recent years, research on pesticide residues has progressed beyond simple post-harvest monitoring to focus on elucidating residue dynamics under field conditions during crop growth and establishing preventive management frameworks at the production stage [1,2,3,4]. This paradigm shift provides essential scientific evidence for developing crop-specific pesticide management strategies by quantitatively determining rate constants and biological half-lives, thereby improving our understanding of residue dissipation behavior during cultivation [1,2].

Recently, the concept of cumulative risk assessment (CRA) has been introduced to complement conventional single-compound assessments by considering combined exposure to groups of pesticides that share a common mode of action [5,6,7]. The reliability of CRA largely depends on the accuracy of fundamental toxicological and exposure parameters, including each pesticide’s dissipation behavior, rate constant, biological half-life, and estimated daily intake (EDI) [5,6]. Therefore, clarifying the dissipation behavior of individual pesticides is a prerequisite for scientifically evaluating cumulative risks at the pesticide-group level [6,7].

Among various pesticide classes, pyrethroid insecticides share a common mode of action characterized by the modulation of voltage-gated sodium channels in nerve cells [8]. Because multiple compounds are frequently detected simultaneously in agricultural commodities, this class serves as a representative target group for CRA. Moreover, due to their similar toxicological properties and exposure patterns, acquiring compound-specific dissipation data is essential for enhancing the accuracy and reliability of cumulative risk assessments [9,10].

Within this class, etofenprox is a non-ester-type insecticide characterized by high contact activity and excellent chemical stability. It is widely used to control aphids, thrips, and leaf beetles during crop cultivation [11]. Consequently, etofenprox has been reported as one of the most frequently detected pesticide residues in leafy vegetables, where it is extensively applied for pest management [12,13].

In general, as crop growth accelerates, the concentration of pesticide residues per unit weight tends to decrease due to the dilution effect associated with biomass expansion [1,2]. However, leafy vegetables such as kale (*Brassica oleracea var. acephala*) and red mustard greens (*Brassica juncea*) possess structural features—including broad leaf surfaces, well-developed cuticular wax layers, and fine trichomes—that enhance the retention and adsorption of pesticides [14,15]. Therefore, despite the dilution effect caused by rapid growth, the surface-retention effect resulting from these morphological characteristics may play a more dominant role, which is an important consideration in understanding the residue dissipation behavior of contact-active and chemically stable insecticides such as etofenprox [14,15,16].

Particularly, kale and red mustard greens are widely consumed in Korea as fresh leafy vegetables used for wrapping and are typically eaten raw without thorough washing or cooking. This consumption pattern limits the reduction in pesticide residues during preparation and increases the potential for direct dietary exposure. Accordingly, evaluating the dissipation behavior and dietary risk of etofenprox in these vegetables is essential to ensure consumer safety and to establish effective field-level management approaches [16,17].

Therefore, in this study, a QuEChERS–LC–MS/MS analytical method was employed to accurately quantify daily changes in etofenprox residues in kale and red mustard greens [18,19]. Based on the obtained residue data, rate constants (*k*) and biological half-lives (t_1_/_2_) were calculated to elucidate residue dissipation behavior. In addition, dietary risk assessments were conducted for both average and high-intake consumer groups to provide a scientific basis for establishing effective pesticide safety management strategies during the cultivation of leafy vegetables.

## 2. Materials and Methods

### 2.1. Chemicals and Reagents

The pesticide standard etofenprox (purity 99.27%) was purchased from HPC Standards GmbH (Cunnersdorf, Germany), and its physicochemical properties are detailed in Table A1. The pesticide product used was Severo (etofenprox 20% emulsifiable concentrate) from Kyung Nong Corporation (Seoul, Republic of Korea), a pesticide registered for use on kale and red mustard greens and selected for compliance with critical Good Agricultural Practice (cGAP). UHPLC-grade acetonitrile (ACN), methanol (MeOH), and water were purchased from J.T. Baker (Avantor Performance Materials Korea Ltd., Suwon-Si, Republic of Korea). Formic acid (HPLC grade, purity 99%) was purchased from FUJIFILM Wako Pure Chemical Corporation (Osaka, Japan), and ammonium acetate from Sigma-Aldrich (St. Louis, MO, USA). QuEChERS extraction pouch (EN Method No. 5982-7650) containing 1 g of sodium chloride (NaCl), 4 g of magnesium sulfate (MgSO_4_), 0.5 g of sodium hydrogen citrate sesquihydrate (Na_2_HCitr·1.5H_2_O) and 1 g of sodium citrate (Na_3_Citr·2H_2_O) and dispersive Solid-phase extraction (d-SPE) (No. 5982-5021) tube containing MgSO_4_ 150 mg and PSA 25 mg were purchased from Agilent Technologies (Santa Clara, CA, USA).

### 2.2. Field Trial

Field trials were conducted under greenhouse conditions at three sites selected in accordance with the MFDS manual for field dissipation studies (MFDS, 2014), ensuring a latitudinal separation of more than 20 km: Icheon (Field 1), Yesan (Field 2), and Pyeongtaek (Field 3) for kale, and Icheon (Field 1), Yesan (Field 2), and Anseong (Field 3) for red mustard greens in the Republic of Korea. Temperature and relative humidity were monitored throughout the trials to confirm the environmental conditions at each location. For kale, temperatures were 15.8–16.2 °C (%RSD 16–20%) and relative humidity was 66–74% (%RSD 14–20%), while for red mustard greens, temperatures were 17.4–20.3 °C (%RSD 12–15%) and relative humidity was 59–74% (%RSD 15–20%). These data indicate that the environmental conditions were comparable across the sites, consistent with the greenhouse conditions. Each field trial consisted of three treated plots and one untreated control plot, each with an area of at least 10 m^2^.

A commercial formulation of etofenprox (Severo^®^, 20% EC; Kyung Nong Co., Seoul, Republic of Korea) was applied twice at a 7-day interval using a knapsack sprayer (Model EL969-1, Perfect L Co., Siheung, Republic of Korea). The sprayer was operated at a pressure range of 0.6–1.0 bar for red mustard greens and 2.5–2.8 bar for kale, with an average spray rate of 1200–1230 mL min^−1^ and 1000–1100 mL min^−1^, respectively. The corresponding spray volume ranged from 0.18 to 0.27 L m^−2^, and the pesticide solution was prepared by diluting 5 mL of formulation in 10 L of water, in accordance with the critical Good Agricultural Practices (cGAP) use pattern rec by the Rural Development Administration (RDA, Jeonju-si, Republic of Korea) (Table 1).

Samples were collected at 0 days (within 2 h after the final application) and at 1, 3, 5, 7, 10, and 14 days after the final treatment. Approximately 1 kg of leaves of commercially marketable size was randomly harvested from each replicate plot. The samples were transported to the analytical laboratory in an ice box, frozen at −70 °C for at least 48 h in a deep freezer, homogenized, and then stored at −20 °C until analysis. All experimental procedures were conducted in accordance with the Manual for Pesticide Residue Tests at the Pre-Harvest Residue Limit [20]. The average fresh weight of individual samples increased during cultivation, ranging from 5.3 to 5.9 g at 0 day to 6.7–7.3 g at 14 days for red mustard greens, and from 5.7 to 5.8 g to 6.2–6.7 g for kale.

### 2.3. Instrumental Conditions

Residue analysis of etofenprox was performed using an AB SCIEX ExionLC™ Series UHPLC (AB SCIEX, Concord, ON, Canada) system coupled with an AB SCIEX QTRAP^®^ 5500 LC-MS/MS system (AB SCIEX, Concord, ON, Canada). Chromatographic separation was performed using a reversed-phase Kinetex C_18_ column (100 mm × 4.6 mm, 2.6 µm) from Phenomenex (Torrance, CA, USA). Mobile phase consisted of (A) 5 mM ammonium acetate and 0.1% (*v*/*v*) formic acid in water and (B) 5 mM ammonium acetate and 0.1% (*v*/*v*) formic acid in MeOH. Isocratic elution was applied with a composition of A/B (10:90, *v*/*v*) at a flow rate of 0.3 mL/min for 10 min. The column oven temperature was set at 35 °C, and the injection volume was 2 µL.

Mass spectrometric analysis of etofenprox was performed using positive electrospray ionization (ESI) and multiple reaction monitoring (MRM). The ion spray voltage was set to +4500 V, the source temperature was maintained at 550 °C, and nitrogen was used as the collision gas. To establish the MRM conditions, a 20 µg/L etofenprox standard solution was injected, the scan mode was employed to select the quantifier and qualifier ions, and the collision energy (CE) was optimized based on sensitivity and selectivity. The highest sensitivity was used as the quantifier ion, and the second-highest sensitivity was used as the qualifier ion. The final MRM conditions are summarized in Table A2. The processing of all data was carried out using AB SCIEX Analyst software (AB SCIEX, version 1.6.3; Framingham, MA, USA). All instrumental parameters and quality control procedures were established in accordance with the SANTE/2021/11312 guidelines [21].

### 2.4. Analytical Method

Sample extraction was performed using the QuEChERS procedure (EN 15662 method) with minor modifications [22]. A homogenized sample (5 g) was mixed with 10 mL of acetonitrile and shaken at 1300 rpm for 1 min using a GenoGrinder (Lab System, Seoul, Republic of Korea). A QuEChERS extraction kit (0.5 g Na_2_HCit·1.5H_2_O, 1 g Na_3_Cit·2H_2_O, 1 g NaCl, and 4 g MgSO_4_) was subsequently added. The mixture was shaken again at 1300 rpm for 1 min and then centrifuged at 4000 rpm for 5 min using a centrifuge (Fleta 5, Hanil Science, Seoul, Republic of Korea). Next, 1 mL of the supernatant was transferred into a d-SPE tube containing 25 mg PSA and 150 mg MgSO_4_, vortexed for 1 min, and centrifuged at 13,000× *g* rpm for 3 min. The final supernatant was filtered through a 0.45 µm PTFE syringe filter (Korea Vaccine, Seoul, Republic of Korea) and analyzed by LC–MS/MS.

Method validation was conducted for selectivity, limits of detection (LOD) and quantification (LOQ), linearity, accuracy, precision, and storage stability. Selectivity was evaluated by comparing chromatograms of blank matrix samples with those of matrix-matched standard solutions, confirming that no interfering peaks were observed at the analyte retention time. The limits of detection (LOD) and quantification (LOQ) were determined using six concentration levels of etofenprox standard solutions (0.002, 0.005, 0.01, 0.02, 0.035, and 0.05 µg/mL), analyzed in triplicate. LOD and LOQ were calculated from the standard deviation of the intercept (σ) and the slope of the calibration curve (S) according to the following equations: LOD = 3.3σ/S and LOQ = 10σ/S. Linearity was evaluated using matrix-matched calibration standards in concentrations ranging from 0.002 to 0.05 µg/mL, which was validated by obtaining coefficients of determination (r^2^) ≥ 0.99. Accuracy and precision were evaluated through recovery experiments by fortifying blank samples with standard solutions at three concentration levels, triplicate: LOQ (0.01 mg/kg), 10× LOQ (0.1 mg/kg), and above the MRL (≥15 mg/kg). The mean recovery (%) and relative standard deviation (RSD) at each level were evaluated for compliance with the acceptance criteria specified in the MFDS manual (2014) and the SANTE/2021/11312 guidelines [20,21]. The residue samples were analyzed within 30 days; however, storage stability for periods exceeding 30 days was evaluated to ensure that etofenprox residues remained stable in samples stored at −20 °C until the analyses were completed.

### 2.5. Calculation of Dissipation Constant and Biological Half-Lives

Dissipation curves were plotted based on the pesticide residue concentrations (mg/kg) measured on each sampling day for kale and mustard greens. Dissipation curves were plotted based on the pesticide residue concentrations (mg/kg) measured on each sampling day for kale and red mustard greens, from which the dissipation rate constant (*k*) was calculated using the derived regression equation, as shown in Equation (1) [23].

(1) $$C_{t}=C_{0}\times e^{-kt}$$

where *C_t_* represents the residue concentration at time (*t*), *C*_0_ is the initial concentration (mg/kg), and *k* denotes the dissipation rate constant. The regression analysis provided the dissipation rate constant (*k*), and the biological half-life (t_1_/_2_) was calculated using Equation (2) [24].

(2) $$t_{1/2}=\frac{\ln2}{k}$$

The regression equations and coefficients obtained from triplicate measurements at each sampling date were evaluated for significance using F-tests and *t*-tests. A 95% confidence interval for the regression coefficients was calculated, and the upper limit of this interval was selected as the dissipation rate constant (*k*) [25,26]. All statistical analyses were performed using Microsoft Excel 2023 (Microsoft Corp., Redmond, WA, USA).

### 2.6. Dietary Risk Assessment

In this study, a dietary risk assessment was conducted to evaluate potential consumer dietary exposure to etofenprox residues through the fresh consumption of kale and red mustard greens. The estimated daily intake (EDI, mg/kg bw/day) was calculated using Equation (3) [27].

(3) $$EDI=\frac{C\times F}{bw}$$

where *C* represents the residue level of the pesticide (mg/kg), *F* is the daily food intake (kg/day), and bw is the body weight (kg).

The acceptable daily intake (ADI) value for etofenprox was established by the Joint FAO/WHO Meeting on Pesticide Residues (JMPR) in 2011. The dietary risk assessments of etofenprox in kale and red mustard greens were evaluated by calculating the ratio of the estimated daily intake (EDI) to the acceptable daily intake (ADI), expressed as a percentage (%ADI), which was calculated using Equation (4) [28,29,30].

(4) $$\%ADI=\frac{EDI}{ADI}\times 100$$

The dietary risk assessment of etofenprox was conducted using residue concentration data obtained from field trials combined with food consumption data (representing both average consumption and high consumption at the 99th percentile) from the Korea National Health and Nutrition Examination Survey (KNHANES, 2022) [31]. For the exposure calculation, the highest residue values observed at 0 days and 7 days (the pre-harvest interval, PHI) after the final application were used. The estimated daily intake (EDI) was calculated based on an average adult body weight of 60 kg, which is the default value recommended by FAO/WHO for dietary exposure assessments [32,33,34,35,36].

## 3. Results and Discussion

### 3.1. Method Validation

**Figure A1:**
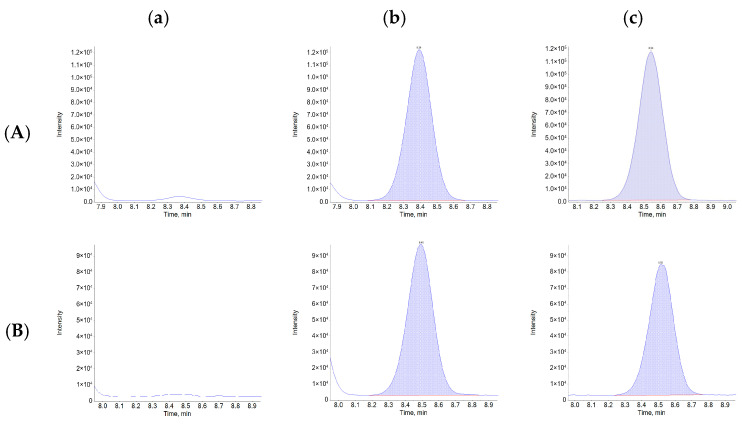
Typical LC–MS/MS chromatograms of etofenprox in (**a**) a blank control, (**b**) a spiked sample (0.01 mg/kg), and (**c**) a field residue sample at 7 days after the final application in (**A**) kale and (**B**) red mustard greens.

The specificity of the analytical method was verified by comparing the chromatograms of blank samples of kale and red mustard greens with those of samples fortified with etofenprox at the 0.01 mg/kg level and field residue samples, with no interfering peaks observed (Figure A1). The limits of detection (LOD) and quantification (LOQ), calculated from the standard deviation of the intercept (σ) and the slope of the calibration curve (S), were determined to be 0.0006 mg/kg and 0.0018 mg/kg, respectively. Considering the sample weight, extraction volume, and injection volume, the analytical method LOQ for matrix samples was established at 0.01 mg/kg [37,38]. Linearity was evaluated by constructing matrix-matched calibration curves at six concentrations (0.002, 0.005, 0.01, 0.02, 0.035, and 0.05 µg/mL) for each crop. The coefficients of determination (r^2^) were ≥0.99 for both matrices, demonstrating a linear relationship within the tested range [39]. The accuracy and precision of the analytical method for etofenprox in both kale and red mustard greens were within an acceptable limit (Table 2). In kale, recoveries ranged from 83.3% to 91.5% with %RSD of 0.7–3.1%, and storage stability was confirmed for 96 days with a recovery of 72.7 ± 3.1%. In red mustard greens, recoveries ranged from 88.9% to 97.1% with %RSD below 6%, and storage stability was confirmed for 64 days with a recovery of 87.8 ± 1.2%. These results indicate that the analytical method is reliable and meets the validation criteria specified in the SANTE and MFDS guidelines.

To evaluate the matrix effect (%ME), the slopes of the calibration curves derived from standard solutions in solvent and matrix-matched extracts were compared according to Equation (5). Ion suppression of −17.5% in kale and −3.3% in red mustard greens, both within |%ME| < 20%, indicated that the matrix effect was low [40].

(5) $$\%ME=(\frac{slope\;of\;matrix-matched\;calibration\;curve}{slope\;of\;solvent-only\;calibration\;curve}-1)\times 100$$

### 3.2. Dissipation Behavior of Etofenprox in Kale and Red Mustard Greens

**Figure 1:**
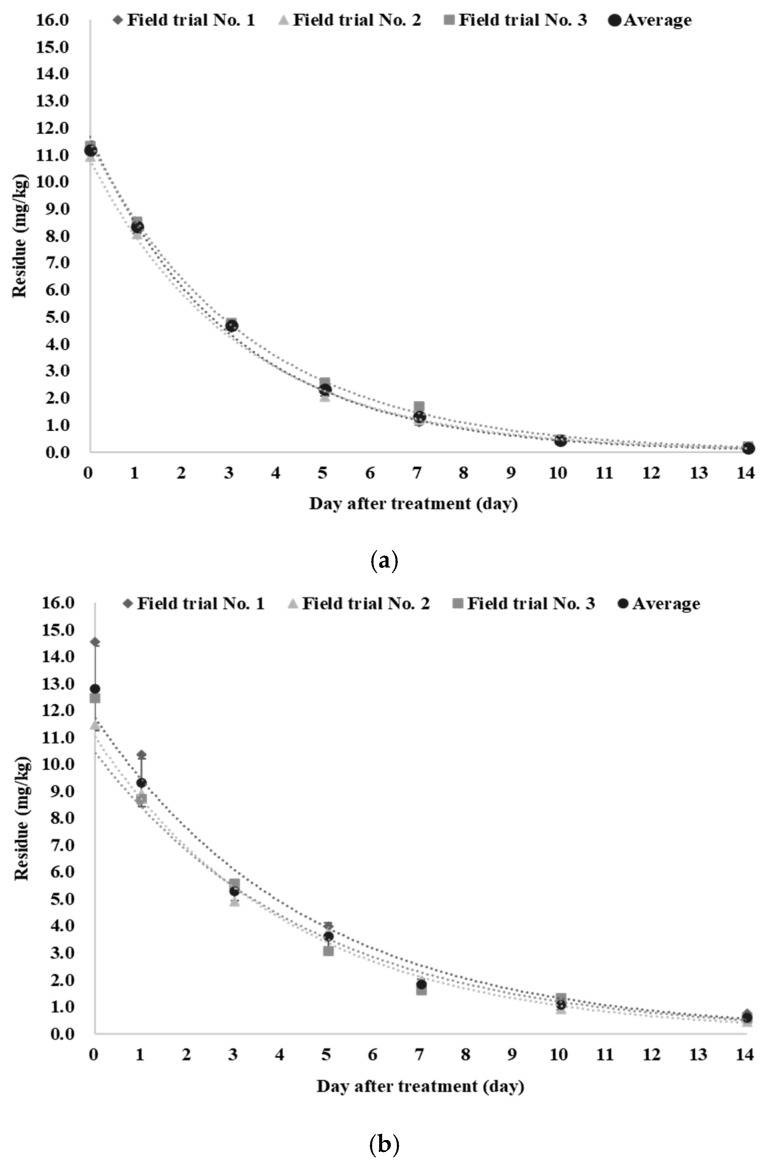
Dissipation curves of etofenprox based on the average residue levels obtained from three field trials. (**a**) Kale; (**b**) Red mustard greens.

The residue concentrations measured on each sampling day after the final application are shown in Figure 1. The average residue concentration at day 0 (within 2 h after final treatment) of red mustard greens showed higher initial residues (12.81 mg/kg) than kale (11.16 mg/kg). The difference in initial residue levels for kale and red-mustard greens is mainly attributed to variations in leaf-surface characteristics, such as the hydrophobicity of epicuticular waxes and the presence of trichomes. In kale, the epicuticular wax layer reduces spray-liquid adhesion and retention, resulting in lower droplet deposition on the leaf surface [41]. In a previous study on rice, leaves that were smooth, lacked trichomes, and contained higher surface wax were shown to retain only small droplets, resulting in reduced spray deposition [42].

Residues in both crops declined to levels below the established MRL (15 mg/kg) before the registered PHI of 7 days and to approximately one-tenth of the MRL by Day 7, indicating a rapid dissipation pattern and a markedly low residue burden at harvest. These residue data may provide a scientific basis for regulatory agencies to evaluate the feasibility of a shorter PHI or a lower MRL, thereby contributing to enhanced consumer safety.

The dissipation of etofenprox residues in both kale and red mustard greens followed first-order kinetics (*C_t_* = *C*_0_ × *e*^−^*ᵏᵗ*), with high coefficients of determination (R^2^ > 0.94) observed in all field trials (Table 3).

The dissipation rate constants (*k*), calculated from the average residue levels determined in field trials, were 0.3118 day^−1^ for kale and 0.2232 day^−1^ for red mustard greens, corresponding to biological half-lives (t_1_/_2_) of 2.2 and 3.1 days, respectively. These values are consistent with previously reported half-lives of etofenprox in other vegetables such as cabbage (2.02–2.38 days) and tomato (2.15 days) [43,44]. Rapid pesticide dissipation has also been observed in leafy vegetables such as spinach, lettuce, and perilla, largely attributable to the growth dilution effect during plant growth [45,46,47,48].

### 3.3. Dietary Risk Assessment

Based on the highest residue levels observed in the field trials at 0 day and at 7 days (PHI) after the final application, dietary exposure was estimated using average and high-intake consumption values. For kale, the average and high-intake consumptions were 0.0064 and 0.0354 kg day^−1^, respectively, whereas for red mustard greens, they were 0.0048 and 0.0330 kg day^−1^, respectively [31]. At 7 days after the final application (PHI), the estimated daily intakes (EDIs) were calculated to be 2.0 × 10^−4^ mg kg^−1^ bw day^−1^ for both crops. When compared with the acceptable daily intake (ADI) of 0.03 mg kg^−1^ bw day^−1^, the corresponding %ADI values for the average consumer group were 0.6% for kale and 0.5% for red mustard greens, respectively, while those for the high-intake group were estimated to be below 4% for both crops (Table 4) [49].

In the worst-case scenario, assuming the highest residue levels detected at 0 days after the final application, the %ADI values increased to 4% for the average consumer group and to 22.3% for kale and 26.6% for red mustard greens in the high-intake group, indicating that dietary exposure to etofenprox through kale and red mustard greens poses a negligible health risk to consumers. In addition, by applying the highest residue concentrations observed on day 0, the acute dietary exposure of adults was estimated and compared with the acute reference dose (ARfD, 1 mg/kg bw). The resulting exposure levels were very low, corresponding to 0.80% of the ARfD for kale and 0.67% for red mustard greens [50,51].

The %ADI values obtained in this study were comparable to those previously reported in Korea, where the %ADI values for pesticide residues in vegetables are typically below 10% of the ADI [52]. These findings were also consistent with the results of the European Food Safety Authority (EFSA, 2021), which reported an average dietary exposure of 0.12% of the ADI for leafy vegetables in Europe [53]. Furthermore, monitoring studies on high-consumption agricultural commodities distributed in Korea have shown that dietary exposures to pyrethroid insecticides are below 1% of the ADI, thereby supporting the findings of this study [54]. Similar results have been observed in other domestic studies. An et al. (2022) reported that the %ADI of etofenprox in Brussels sprouts was 0.81% [55], while Kim et al. (2025) demonstrated that residue reduction through washing and thermal processing decreased the %ADI for cereal-based products to below 1% [56]. In general, both domestic and international research consistently report low dietary risks associated with pyrethroid residues. Yi et al. (2020) found that dietary risks from pesticide residues in vegetables consumed in Korea were generally low, with %ADI values well below health-concern thresholds [57]. Similarly, Li et al. (2023) reported cumulative hazard indices for pyrethroids in fruits and vegetables ranging between 0.004% and 0.200%, indicating negligible chronic dietary risk [58]. The cumulative %ADI (<0.3%) calculated for etofenprox in kale and red mustard greens was relatively low, indicating minimal cumulative dietary risk. These findings suggest that the EDI and %ADI data from this study can provide a practical basis for future cumulative risk assessment (CRA) of pyrethroid insecticides.

In further studies, we plan to conduct stepwise studies on additional pyrethroid pesticides to progressively advance a cumulative exposure assessment framework for the pyrethroid class. Furthermore, we will incorporate residue levels after washing to provide a more realistic dietary exposure assessment for the fresh consumption of leafy vegetables.

## 4. Conclusions

This study investigated the dissipation pattern of etofenprox residues after pesticide application under field cultivation conditions in kale and red mustard greens. The initial residues after pesticide application and those at the pre-harvest interval (PHI) were both below the Korean maximum residue limits (MRLs) in kale and red mustard greens. Based on these residue levels, the dietary risk assessment showed that the %ADI values were very low, ranging from 0.01% to 0.04% for the average-consumption group and from 0.03% to 0.27% for the high-consumption group at 7 and 0 days after pesticide application, respectively. Dietary risk assessment indicated that the cumulative %ADI of etofenprox in kale and red mustard greens was below 0.3%, even in the high-consumption group, indicating a very low cumulative dietary risk. Therefore, the findings of this study provide a scientific basis for cumulative risk assessment (CRA) of pyrethroid insecticides and will contribute to the refinement of multi-residue dietary exposure evaluation in future food safety assessments.

## Figures and Tables

**Table 1:** Critical GAP use pattern of etofenprox for kale and red mustard greens.

Formulation	A.I (%) ^a^	Dilution	Application No.	Application Interval (Days)	Pre-Harvest Intervals (Days)
Emulsion Concentrate	20	2000	2	7	7

^a^ Active Ingredient.

**Table 2:** Recovery and storage stability of etofenprox in kale and red mustard greens.

Crop	Validation Parameters	Spiked Level (mg/kg)	Recovery (%), *n* = 3	
			Mean ± SD ^a^	%RSD ^b^
Kale	Accuracy and precision	0.01	83.3 ± 0.6	0.7
		0.1	91.5 ± 1.7	1.8
		15	88.7 ± 2.8	3.1
	Storage stability (96 days)	1	72.7 ± 3.1	4.3
Red mustard greens	Accuracy and precision	0.01	94.3 ± 0.6	0.6
		0.1	97.1 ± 0.6	0.7
		20	88.9 ± 5.2	5.9
	Storage stability (64 days)	1	87.8 ± 1.2	1.4

^a^ Standard deviation; ^b^ Percentage relative standard deviation, calculated as (SD/Mean recovery) × 100.

**Table 3:** Dissipation regression analysis and biological half-lives of etofenprox in kale and red mustard greens.

Crops	Field No.	Dissipation Regression Curve ^a^	Biological Half-Life (Day)	Dissipation Rate Constant ^b^
Kale	1	y = 11.6516e^−0.3294x^ (R^2^ = 0.9982)	2.1	0.3154
	2	y = 10.8132e^−0.3135x^ (R^2^ = 0.9981)	2.2	0.2942
	3	y = 11.4640e^−0.2965x^ (R^2^ = 0.9990)	2.3	0.2641
	Average	y = 11.2928e^−0.3118x^ (R^2^ = 0.9992)	2.2	0.2926
Red mustard greens	1	y = 11.7231e^−0^.^2194x^ (R^2^ = 0.9773)	3.2	0.1656
	2	y = 11.0118e^−0.2370x^ (R^2^ = 0.9945)	2.9	0.2149
	3	y = 10.4248e^−0.2175x^ (R^2^ = 0.9828)	3.2	0.1748
	Average	y = 11.0408e^−0.2232x^ (R^2^ = 0.9885)	3.1	0.1889

^a^ Significant at *p* < 0.05 by the F-test; ^b^ Significant at *p* < 0.05 by the *t*-test.

**Table 4:** Estimated daily intake and percentage of acceptable daily intake of etofenprox in kale and red mustard greens.

Crop	Sampling Intervals (Days)	HR ^a^ (mg/kg)	Intake Group	Daily Intake (kg/Day)	EDI ^b^ (mg/kg·bw/Day)	ADI ^c^ (mg/kg·bw/Day)	%ADI
Kale	0	11.34	High Average	0.0354 0.0064	0.0067 0.0012	0.03	22.3 4.0
	7	1.70	High Average	0.0354 0.0064	0.0010 0.0002		3.3 0.6
Red mustard greens	0	14.53	High Average	0.0330 0.0048	0.0080 0.0012		26.6 3.9
	7	1.99	High Average	0.0330 0.0048	0.0011 0.0002		3.6 0.5

^a^ Highest residue from the field trials data; ^b^ Estimated daily intake; ^c^ Acceptable daily intake.

## Data Availability

The original contributions presented in this study are included in the article. Further inquiries can be directed to the corresponding author.

## Appendix A

**Table A1:** Physicochemical properties and chemical structure of etofenprox.

IUPAC Name	2-(4-Ethoxyphenyl)-2-Methylpropryl 3-Phenoxybenzyl Ether
Molecular formula	C_25_H_28_O_3_
Molecular weight (g/mol)	376.49 g/mol
Log Pow	6.9 (20 °C)
Vapor pressure	8.13 × 10^−4^ mPa (25 °C)
Water Solubility (mg/L, 20–25 °C)	0.0225
Structure	
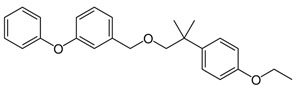

**Table A2:** Multiple reaction monitoring (MRM) conditions for etofenprox.

Exact Mass	MW ^a^	Precursor Ion (*m/z*)	Product Ion (*m/z*)	CE ^b^	RT (min) ^c^
376.2	376.5	394.3	177.1 ^d^	20	8.39
			107.1 ^e^	61	

^a^ Molecular Weight; ^b^ Collision Energy; ^c^ Retention time; ^d^ Quantitation ion; ^e^ Confirmation ion.
